# Does tomosynthesis increase confidence in grading the suspicious appearance of a lesion? An audit of cancers diagnosed in the assessment clinic using tomosynthesis: initial experience at Avon Breast Screening Unit

**DOI:** 10.1186/bcr3787

**Published:** 2015-11-05

**Authors:** Gillian Clark, Alexandra Valencia

**Affiliations:** 1 Avon Breast Screening Unit, Bristol Breast Care Centre, North Bristol NHS Trust, Bristol, UK

## Introduction

Tomosynthesis is a new technology that is being used increasingly to evaluate the breast for assessment in the UK. It has, however, been approved as a screening tool in the United States, Canada and several European countries. We implemented tomosynthesis in the Assessment Clinic at Avon Breast Screening Unit (ABSU) last year as recommended by the NHSBSP. A retrospective audit of 134 consecutive cancers diagnosed from 9 June 2014 to 31 December 2014 was performed. The aim was to evaluate whether tomosynthesis gives additional information to increase the grading of mammographic features of a lesion seen on initial screening mammography and increase the assessor's confidence.

## Result

A total of 134 cancers were reviewed. Sixty-six lesions were graded the same on screening mammography and the assessment tomosynthesis. Thirty-six were M5 lesions at screening and assessment. Thirty M3 or M4 lesions remained unchanged. One patient had an M3 lesion that was downgraded. Three patients had incidental cancers found on ultrasound. Sixty-four lesions were upgraded with tomosynthesis. Forty-four of 64 M3 or M4 lesions were upgraded to tomosynthesis 5. Twenty of 64 were upgraded from M3 to tomosynthesis 4. The morphology of the lesions upgraded was spiculated 30/64, 7/64 distortions and 7/64 ill-defined densities. Thirty-one of 44 tomosynthesis 5 lesions measured 10 mm or less.

## Conclusion

Tomosynthesis is excellent at showing the spiculate nature of lesions, upgrading the appearance of a lesion from M3 and M4 to tomosynthesis 5 which increases the assessor's confidence during the assessment clinic. It is also excellent in helping identify small suspicious lesions of 10 mm or less. However, ultrasound should always be performed in addition to tomosynthesis as lesions may rarely be downgraded.

